# Consequences for Pancreatic β-Cell Identity and Function of Unregulated Transcript Processing

**DOI:** 10.3389/fendo.2021.625235

**Published:** 2021-03-08

**Authors:** Seyed M. Ghiasi, Guy A. Rutter

**Affiliations:** Section of Cell Biology and Functional Genomics, Division of Diabetes, Endocrinology and Metabolism, Department of Metabolism, Digestion and Reproduction, Faculty of Medicine, Imperial College London, London, United Kingdom

**Keywords:** β-cell, insulin secretion, transcript, nonsense-mediated decay, RNA decay, RNA processing

## Abstract

Mounting evidence suggests a role for alternative splicing (AS) of transcripts in the normal physiology and pathophysiology of the pancreatic β-cell. In the apparent absence of RNA repair systems, RNA decay pathways are likely to play an important role in controlling the stability, distribution and diversity of transcript isoforms in these cells. Around 35% of alternatively spliced transcripts in human cells contain premature termination codons (PTCs) and are targeted for degradation *via* nonsense-mediated decay (NMD), a vital quality control process. Inflammatory cytokines, whose levels are increased in both type 1 (T1D) and type 2 (T2D) diabetes, stimulate alternative splicing events and the expression of NMD components, and may or may not be associated with the activation of the NMD pathway. It is, however, now possible to infer that NMD plays a crucial role in regulating transcript processing in normal and stress conditions in pancreatic β-cells. In this review, we describe the possible role of Regulated Unproductive Splicing and Translation (RUST), a molecular mechanism embracing NMD activity in relationship to AS and translation of damaged transcript isoforms in these cells. This process substantially reduces the abundance of non-functional transcript isoforms, and its dysregulation may be involved in pancreatic β-cell failure in diabetes.

## Introduction

Diabetes mellitus currently affects ~460 m adults worldwide and its incidence is expected to exceed 700 m by 2045 (1). Both T1D and T2D – the latter being the predominant form – involve defective pancreatic β-cell function, and a contribution of inflammatory processes which is most acute in the former. β-Cell death is marked in T1D: typically >80% of the cell mass is lost, though this figure is lower in some patients, particularly those with later disease onset (2), but more limited in T2D (3) where dysfunction predominates (4). The molecular mechanisms involved in β-cell dysfunction and loss in both settings are only partly understood.

Insults associated with diabetes including challenge with inflammatory cytokines, hyperglycaemia and gluco(lipo)toxicity downregulate selected β-cell specific transcripts and up-regulate others (4–6). This is followed by a profound up-regulation of mRNA surveillance systems (7). The up-regulation of splicing factors, and of proteins involved in pre-mRNA processing, gives rise to alternative splicing (AS) events, which in turn deregulate the balance and turnover of transcript isoforms. Interestingly, most human genes exhibit alternative splicing, but not all alternatively spliced transcripts are translated into functional proteins and so targeted for degradation. This varies in a cell-specific manner and depends on the capacity of cells to cope with damaged transcripts (7–9).

At least four types of mRNA decay pathways have been studied in mammalian cells and which scrutinize transcript quality: nonsense-mediated decay (NMD), Staufen1 (STAU1)-mediated mRNA decay (SMD), no-go decay (NGD), and nonstop-decay (NSD) (10–12). Here, we focus on how the NMD pathway interacts with alternative splicing to regulate transcript isoform expression. We also consider how as its deregulation may contribute to β-cell dysfunction, vulnerability, and destruction in diabetes.

## Alternative Splicing (AS); Implications In β-Cells

**Figure 1:**
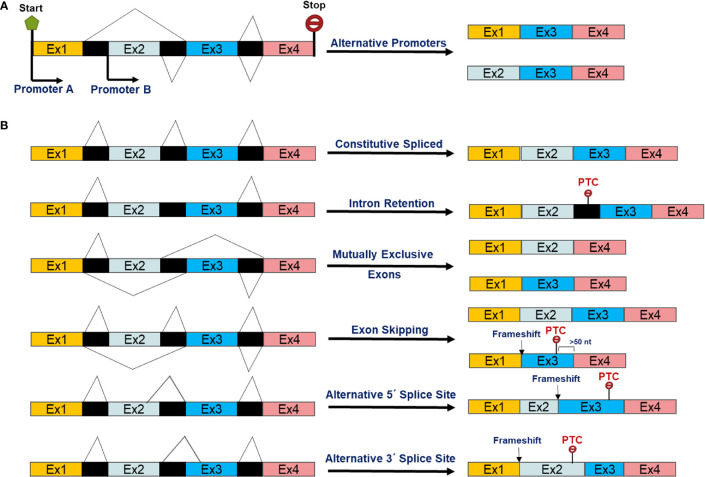
Scheme of alternative transcript isoforms as a result of **(A)** alternative promoters and post-transcriptional alternative splicing events of pre-mRNAs **(B)**.

Non-coding interspaced sequences, namely introns, are removed by the splicesome, a dynamic RNA-protein complex, during transcription from precursor-mRNAs (13, 14). Around 90%–95% of human transcripts are thought to leave the nucleoplasm as pre-mRNAs that need further splicing and processing to become a mature mRNA (15). Alternate (or alternative) isoforms created either by switching the usual promoter to alternative promoter of a gene and/or pre-mRNA alternative splicing (**Figure 1A**). Alternative splicing, an evolutionarily post-transcriptional pre-mRNA processing process, produces multiple distinct transcript variants of most human genes (16, 17). The mechanisms and biology of splicing and alternative splicing have been extensively reviewed (18–20). As described previously (15) and in **Figure 1B**, AS can involve inclusion or skipping (exclusion) of an exon, mutually exclusive exons, alternative 5′ donor splice sites, and alternative 3′ acceptor splice sites and intron retention in mRNAs (15). AS is a consequence of interactions between RNA binding proteins (RBPs) and splicing regulatory elements (SREs) in pre-mRNAs. The Serine Arginine rich proteins (SRs) are a family of constitutive or regulatory RNA binding proteins recognize pre-mRNA SREs through interaction with the N-terminal and C-terminal domains enriched with arginine (R) and serine (S) sequences. These then interact with other proteins and/or SREs to enhance splicing by recruiting the spliceosome (21). On the other hand, the second class of RBPs, members of the heterogeneous nuclear ribonucleoprotein (hnRNP) protein family, have been shown to antagonize SR functions by competing for binding to exonic splicing enhancers (ESE) or intronic splicing suppressors (ISS) (21, 22).

Alternative splicing is observed in all human tissues, but has been most extensively studied in neurons (23, 24). Thus, aberrant alternative splicing of the pre-mRNAs encoding calcium signalling transducers affects neuronal function and causes neurodegenerative diseases (25–27). In the past decade, pancreatic β-cell transcriptomic analyses have revealed differential expression profiles of the RBPs and splicing factors which are abundantly expressed in neurons, and whose genetic ablation can lead to impairments in insulin secretion and reduced β-cell viability (28–30).

It is now established that alternative splicing plays an important role in β-cell function and viability. Glucose, a major regulator of pancreatic β-cell function (31), strongly affects insulin gene expression, biosynthesis, and secretion, through multiple mechanisms including changes in transcription, pre-mRNA alternative splicing, translation and mRNA stability (32–34). Insulin intron-2-containing pre-mRNA levels increased six-fold within an hour of a human islet exposure to high glucose, whereas increases in mature mRNA did not occur before 48 h of exposure (35), suggesting that substantial of insulin production is exerted at the level of pre-mRNA alternative splicing. Another study (36) showed that alternative splicing of the insulin receptor is regulated by insulin signalling and modulates β-cell survival in an autocrine pathway involving insulin secretion, binding to and activation of insulin receptors in human and mouse islets and in clonal MIN6 cells.

Several lines of evidence support the importance of regulated AS in inflammatory stresses in pancreatic β-cells, as reviewed previously (28, 37). The pro-inflammatory cytokines interleukin-1β (IL-1β) and interferon-ɣ (IFNɣ) upregulate >30 splicing factors, affecting alternative splicing of 35% of genes in the human islet transcriptome (9, 37). Genetic manipulation of several RBP candidates involved in alternative splicing, as listed in **Table 1**, impaired insulin secretion and sensitised β-cells to basal and/or cytokine-induced toxicity. These changes may reflect deregulation of transcript isoforms encoding anti/pro-apoptotic proteins as well as those annotated in exocytosis and secretory pathways in pancreatic β-cells (28, 40, 41). Finally, genome-wide RNA sequencing revealed that three transcript isoform variants of *CD137* were associated with T1D development in the NOD mice (42).

**Table 1:** Examples of the function of alternative splicing regulators in β-cells.

	Transcript targets in β-cells	Knockout/downregulation phenotypes in β-cells	References
NOVA1	INSR, PLCβ1, SNAP-25	• Increase in basal and cytokine-induced cell death • Impairment of GSIS	(38)
NOVA2	Not identified	• Increase in basal and cytokine-induced cell death • no effect on GSIS	(39)
RBFOX1 (A2BP1)	Gsn, Cacna1c,	• No effect on apoptosis • Increase in GSIS	(39)
RBFOX2 (RBM9)	Not identified	• No effect on apoptosis • Increase in GSIS and insulin content	(39)
ELAVL4	Not identified	• Increase in apoptosis in basal condition, but decrease in cytokine-induced apoptosis • no effect on GSIS	(39)
SRSF6 (SRp55)	Pro-apoptotic Bcl2 proteins, e.g., BCL2L11, BAX, BOK, DIABLO, BCLAF1 JNK pathway transducers, e.g., MAP3K7, JNK1, and JNK2	• Increase in apoptosis in basal condition • Impairment of GSIS • Induction of ER stress	(30)

At present, there is little direct evidence that genetic variants in the genes involved in components of the NMD pathway are involved in altered T2D risk (43). Nevertheless, alternative splicing of genes which *are* implicated may be relevant. For example, variants in the Transcription factor 7–like 2 (*TCF7L2*) gene are strongly associated with T2D risk in man (44–47). Mapping of *TCF7L2* splice variants revealed a specific pattern in pancreatic islets, with variants carrying exons 4 and 15 correlated with glycated haemoglobin A1c (HbA1c) (48). The presence of deleterious *TCF7L2* splice variants (i.e., exons 13-16) was also suggested to be a mechanism of β-cell failure in T2D mouse models (49). Although deletion of *TCF7L2* selectively in the pancreas (50) or β-cell (51) in mice lowers β-cell function, increased levels of *TCF7L2* mRNA are associated with elevated diabetes risk in man (52, 53), with the latter study reporting increased levels of the 3’ exon (and 15 o 18 exons overall) in islets of carriers of the risk rs7903146 allele. However, and as previously discussed (51), risk allele-dependent alternative splicing of the *TCF7L2* gene in β-cells may affect the inclusion of a “CRARF” motif in the expressed protein and, as such, may impact the transcriptional activity of this factor (i.e., lowered transcriptional activity despite an increased overall transcript load).

The circadian clock has recently been shown to modulate synchronicity of insulin secretion in dark-light phases by regulating the alternative splicing of pre-mRNAs coding for proteins involved in insulin biosynthesis and exocytosis in primary mouse β-cells. The circadian clock core transcription factors CLOCK and BMAL1 autonomously determine oscillatory regulation of ~27% of the β-cell transcript isoforms corresponding to genes coding for proteins that are involved in the assembly, trafficking, and fusion of secretory vesicles at the plasma membrane (54). Disruption of the *CLOCK* and *BMAL1* genes perturbs rhythmic genome-wide alternative splicing of pre-mRNAs encoding regulators of insulin biosynthesis and secretion in murine insulin-producing cell lines and primary β-cells (41, 54). A later exploration of the underlying mechanisms revealed that thyroid hormone receptor-associated protein 3 (THRAP3), an RNA-binding protein, modulates circadian clock-dependent alternative splicing of calcium/calmodulin-dependent serine protein kinase (Cask) and MAP kinase-activating death domain (Madd). Consistent with findings of exon skipping due to circadian clock perturbation, CRISPR-Cas9-mediated deletion of exons-11 and -26 of *Cask* and *Madd* pre-mRNAs, respectively, impairs insulin secretion in murine insulin-producing β-cells (41). *Madd* knockout mice developed hyperglycaemia associated with impaired insulin secretion in mice (55).

Alternatively spliced transcript variants can produce functionally different protein isoforms with altered amino acid sequences and protein domains, resulting in modification of activity. This, in turn, may drive alterations in protein localization, interaction with binding partners or post-translational polypeptide processing (56, 57). A substantial number of alternatively spliced variants contain a premature termination codon (PTC) or other mRNA “discrediting” features such as an upstream open reading frame (uORF), long 3’ untranslated region (UTR) or the retention of introns after stop codons (58, 59). Any of these could potentially render the mRNA a target forf nonsense-mediated decay (60–62). Whether these isoforms are (mis-)expressed in pancreatic β-cells in diabetes – for example as a result of inflammatory or metabolic stresses – remains to be explored.

## The NMD Pathway: Biology and Emerging Role in β-Cells

The nonsense-mediated decay pathway, originally identified as an RNA surveillance mechanism, eliminates aberrant RNAs harbouring PTCs (63). Computational and experimental results indicate that roughly a third of reliably inferred alternative splicing events in humans result in mRNA isoforms that harbour a PTC (64, 65). PTCs can arise in cells through various mechanisms: germline or somatic mutations in DNA; errors in transcription; or post-transcriptional mRNA damage or errors in processing, notably including alternative splicing (66). PTCs have been implicated in approximately 30% of all inherited diseases, indicating that the NMD pathway plays a vital role in survival and health (11, 67). Failure to recognize and eliminate these unproductive transcripts seems likely to result in the production of truncated dysfunctional proteins that directly perturb cell function or lead to an accumulation of misfolded proteins that accumulate in the ER to cause ER stress.

**Figure 2:**
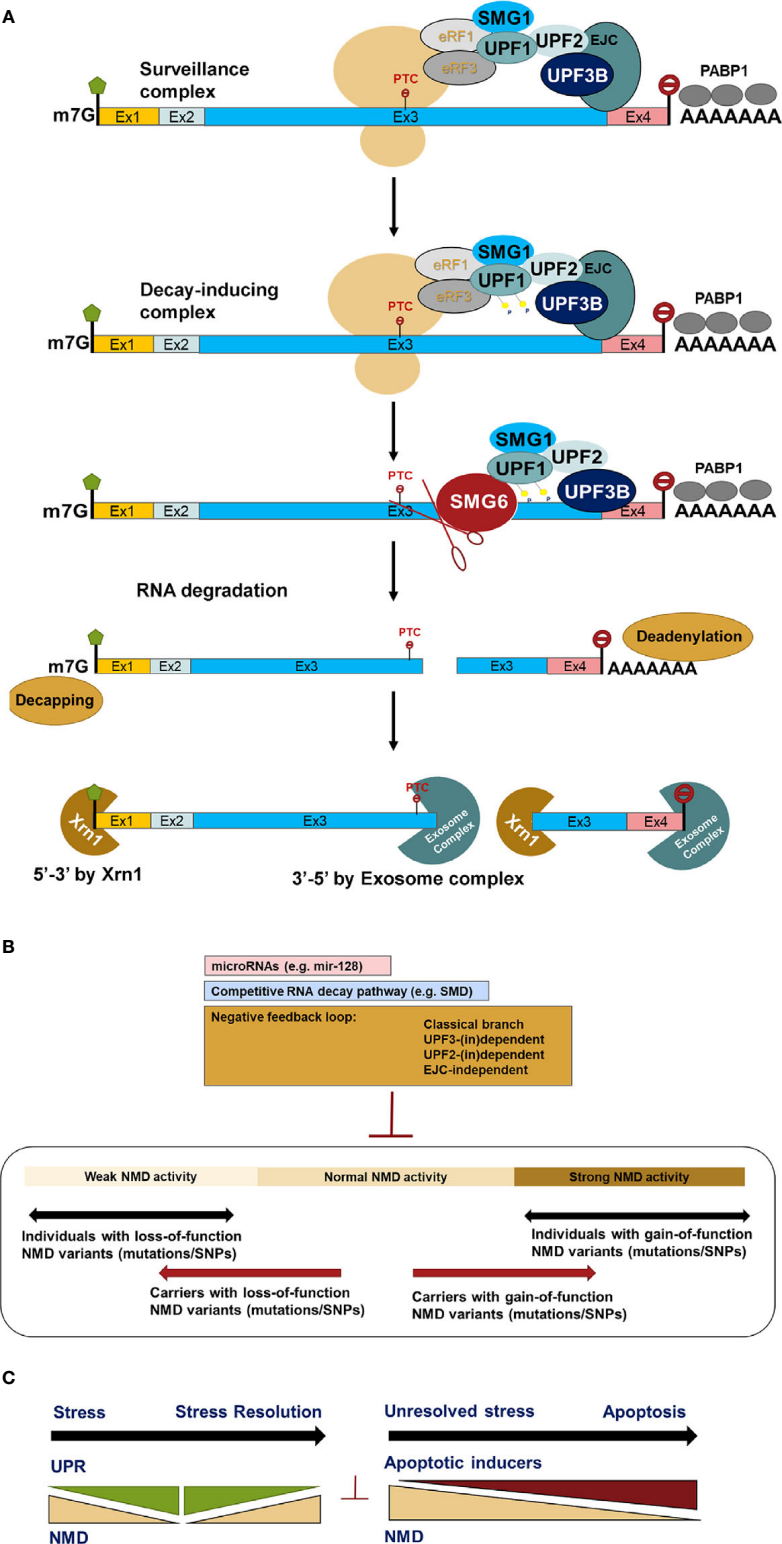
Simplified canonical model of NMD pathway activation **(A)** in human cells which is controlled by three different ways so far and yet to be identified **(B)**. Inter-individual variation of NMD efficiency due to transcript variants modifies the presentation of clinical phenotypes and response to the PTC read-through drug PTC124 treatment **(B)**. At early ER stress, UPR suppresses NMD to provide maximum capacity of UPR proteins replenishment. Once ER stress is being resolved, the UPR is downregulated, while NMD is supposed to return to its normal activity to eliminate unproductive transcript isoforms, thereby leading to further downregulation of the UPR as UPR transcripts isoforms are NMD specific targets. Unresolved ER stress induces apoptosis which in turn suppresses NMD pathway to execute the cell to avoid deleterious outcomes **(C)** (59, 68, 69, 71).

The human NMD machinery is complex and involves multiple proteins including Upf1, Upf2, Upf3a, Upf3b, Smg1, Smg5, Smg6, And Smg7 (See **Table 2**). Together, these are responsible for the detection and decay of PTC-containing transcripts (**Figure 2A**). ATPase-dependent RNA helicases play a central role in NMD activity. Thus, the ability of UPF1 to selectively target PTC-containing mRNAs depends on its ATPase and helicase activities (59, 72). Additionally, activation of NMD requires an interaction between Upf1 and protein partners on the targeted mRNA. These partners consist of UPF2 that forms a bridge between Upf1 and Upf3, forming the Upf1–Upf2–Upf3 complex (66, 73). However, in addition to canonical NMD pathway in which all key NMD components function on target transcripts, NMD is also activated (in)dependent of some of its key factors including Upf2 and Upf3 with a cell-type specific manner. Thus, NMD should be seen as a “branched pathway”, with the different branches defined by autoregulatory feedback loops (**Figure 2B**) (58, 66, 74).

**Table 2:** Characteristics of core machinery and effective NMD components.

NMD component	MW (kDa)	Alternative names	Localization	Direct NMD interactors	Functions in NMD
UPF1 (an ATP-dependent RNA helicase)	123	NORF1, RENT1, smg-2	shuttling to nucleus, but mainly in cytoplasm	UPF2, SMG1, SMG6, SMG7	ATP-dependent helicase, RNA binding protein; regulated by phosphorylation; direct binding to eRFs, PNRC2, and decapping factors
UPF2	148	RENT2, smg-3	Perinuclear (cytoplasmic)	UPF1, SMG1, UPF3	Regulates UPF1 helicase activity; stimulates SMG1 kinase activity; establishes a physical link between UPF1 and UPF3
UPF3A	55	RENT3A, UPF3	shuttling to nucleus, but mainly in cytoplasm	UPF2, EJC	Establishes a physical link between UPF1-UPF2 and the EJC; EJC-independent function unknown
UPF3B	56	RENT3B, UPF3X	shuttling to nucleus, but mainly in cytoplasm	UPF2, EJC	Establishes a physical link between UPF1-UPF2 and the EJC; EJC-independent function unknown, promotes UPF1 phosphorylation; UPF3B- independent is the NMD branch
SMG1 (A PI3‐kinase‐like kinase)	410	ATX, LIP	Cytoplasm and nucleus	UPF1, UPF2, SMG8, SMG9	Phosphorylates UPF1
SMG5	114	EST1B	Cytoplasm and nucleus	UPF1, SMG7	Forms a complex with SMG7; recruits PP2A for UPF1 dephosphorylation; provides additional binding affinity to phosphorylated UPF1
SMG6 (A endoribonuclease)	160	EST1A	Cytoplasm and nucleus	UPF1, EJC	Promotes UPF1 dephosphorylation; directly degrade transcripts at vicinity of the PTC
SMG7	122	EST1C	Cytoplasm	UPF1, SMG5	Forms a complex with SMG5; required for SMG5/7 binding to phosphorylated UPF1; recruits POP2 for mRNA deadenylation
SMG8	110	FLJ10587, FLJ23205	Not identified	SMG1, SMG9	Regulation of SMG1 kinase activity; induces inactivating conformational changes in SMG1
SMG9	58	FLJ12886	Not identified	SMG1, SMG8	Regulation of SMG1 kinase activity; required for SMG1 complex

This table is extracted from these review articles (58, 68–70).

Upf1 promiscuously binds to both NMD-targeted and non-targeted mRNAs undergoing translation (75). On PTC-containing mRNAs, Upf1 and its associated phosphoinositide 3-kinase (PI3K)-like kinase, Smg1 act as a clamp to bind to eukaryotic releasing factors 2 and 3 (eRF2 and eRF3) to form a surveillance complex. The exon-exon junctions at least 50 nucleotides downstream of stop codons possesses a nucleation point where the EJC, Upf2, and Upf3b bind as a foundation of a decay-inducing complex whose Interaction with the surveillance complex triggers Upf1 phosphorylation, dissociation of eRF1 and eRF3, and conformational remodelling of NMD. This adopts Upf1 activity to resolve mRNA secondary structure by its helicase activity, allowing access to the mRNA of the NMD effector proteins Smg5-7 (59, 72–77). The decay of targeted transcripts then takes place through the following steps: recruitment of the endoribonuclease Smg6, which catalyses PTC-proximal mRNA cleavage, producing 5ʹ and 3ʹ cleavage fragments that are degraded by exoribonucleases (73, 78, 79); recruitment of the Smg5–Smg7 heterodimer, which bridges an interaction with the carbon catabolite repressor protein 4 (CCR4)-NOT deadenylase complex, thereby shortening the poly(A) tail to stimulate mRNA decapping by the general decapping complex (80–82); recruitment of the decapping enhancer Proline-rich nuclear receptor co-activator 2 (PNRC2), possibly in a complex with SMG5, which recruits the general decapping complex (83); and/or direct recruitment of the general decapping complex (73, 79, 84). Decapped mRNA is in turn degraded from the 5΄-end by the Xrn1 exoribonuclease and from the 3’ terminus by the Dis3L1 and/or Dis3L2 exosome complex (85, 86). Xrn1 has also been shown to promote general 5΄-3΄ co-translational mRNA decay following the last translating ribosome (85). These degradation pathways are not mutually exclusive, and their balance varies depending on the particular mRNA or organism (73, 86).

NMD pathways have been studied in different human and mouse tissues, and most recently by one of us (SMG) in human and rodent islets (7). These studies revealed that NMD components are differentially up/down-regulated by inflammatory cytokines and glucolipotoxicity. Genetic suppression of the key NMD component Smg6, an endoribonuclease which cleaves NMD targeted transcripts at the proximate location of the PTC (**Table 2**), alleviated cytokine-mediated toxicity associated with increased insulin biosynthesis and glucose–induced insulin secretion in INS-1 cells, a rat insulinoma-derived insulin-producing cell line. This study also revealed that nitroxidative stress is mechanistically involved in cytokine-mediated up-regulation of NMD components, since chemical inhibition of inducible nitric oxide (iNOS) by N-methyl-L-argenine (NMA), normalized cytokine upregulation of the NMD components in INS-1 cells (7). Whether this up-regulation of NMD components culminates in activation of the pathway remains to be elucidated.

Buffering mechanisms appear to have evolved in a cell-type specific manner to control NMD activity, reflecting its important role in regulating normal transcripts as well as eliminating unproductive transcript isoforms. Three different mechanisms include, firstly, microRNAs such as mir-128 targeting NMD component transcripts; another RNA decay pathway called STAU1-mediated mRNA decay (SMD) whose the RNA-binding protein Staufen 1 competes with Upf1 of the NMD, and auto-regulatory feedback (**Figure 2B**) (87–90). The feedback loop has been reported to be exerted at both the mRNA and protein levels of the NMD factors. At the mRNA level, NMD controls the rate-limiting mRNA levels of its components. At the protein level, control is also exerted by protein stabilization of proteins involved in regulation such as Upf3a, an RNA-binding protein suppressing NMD activity (91, 92). It is therefore is not surprising that different branches of the NMD pathway have different efficiency profiles in different tissues (58, 93). Nevertheless, it will be important to determine what branches of NMD control pathway activity in stress conditions in primary pancreatic β-cells.

**Figure 3:**
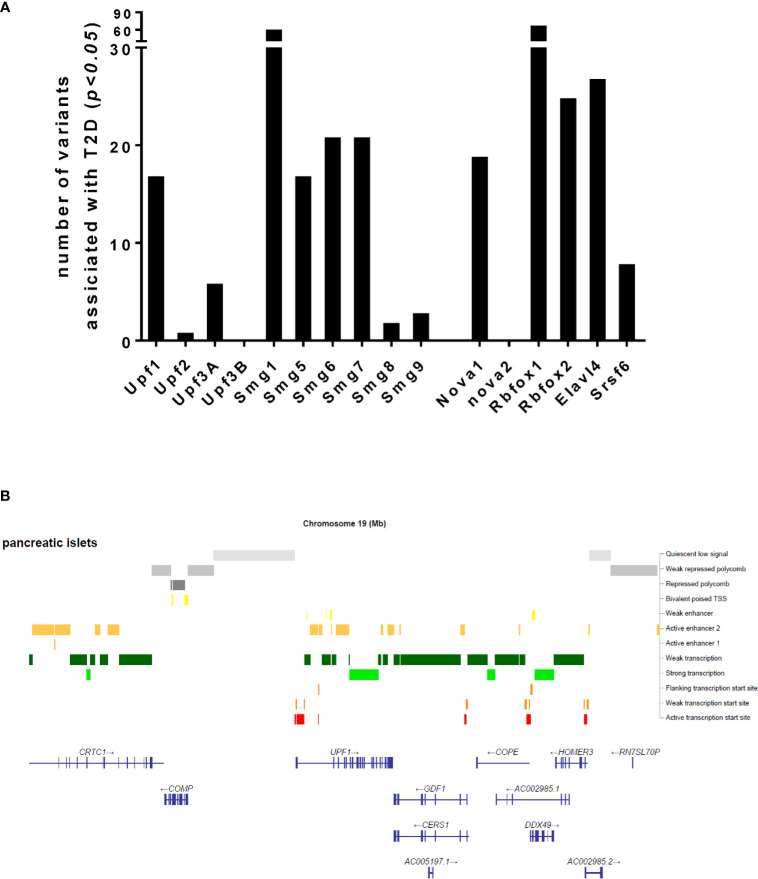
Number of transcript variants of the NMD pathway components and alternative splicing regulators associated with type 2 diabetes **(A)** and an ATAC-sequencing analysis for chromatin accessibility of the Upf1 gene **(B)**, extracted from publicly available type 2 diabetes datasets.

Could NMD transcript variations such as genetic mutations, SNPs and environmental factors affecting NMD activity be associated with cause and development of diabetes? Considering the emerging role of NMD in regulating transcript processing in pancreatic β-cells, we sought to understand whether transcript variants of NMD components are associated with T2D. Our interrogation of publicly-available GWAS data (type2diabetesgenes.org) reveals that an accumulated list of the NMD transcript variants with surprisingly high burden of natural loss-of-function variation including stop-gained, essential splice, and frameshift variants are significantly associated with T2D (*p<0.05*) (**Figure 3A**). In addition, chromatin analysis using ATAC-sequencing, a popular method for determining chromatin accessibility across the genome, indicates high chromatin accessibility to enhancers and subsequent strong transcription upon the key NMD components, e.g., Upf1 and Upf2 and Upf3a (**Figure 3B**).

## Transcript Processing in the β-Cell Transcriptome

The integrity and accuracy of transcript processing is likely to be crucially important to shape the transcriptome of the β-cell and, in turn, to meet the physiological demands and pathophysiological challenges it faces.

In addition to degrading PTC-containing transcripts (i.e., “unproductive” transcripts) (63), NMD is also involved in normal physiology and in the transcriptional regulation of normal transcripts (i.e., “productive” transcripts), functioning as a fine-tuning mechanism of gene expression (59, 71). In fact, early embryonic lethality of mice depleted of the NMD factors *Upf1, Upf2, Upf3a, Smg1*, and *Smg6* suggests that NMD is important for normal development and growth of the cell (60, 94–97). Whether impaired transcript processing due to deregulated NMD pathway may induce the dedifferentiation of β-cells, as may occur in T2D (98) and possibly T1D (99), or hinders interactions between β- with other cell types in the islet (100, 101) are important questions.

In T2D, a compensatory increase in insulin secretion in response to insulin resistance can stimulate a sequence of “stressful” events in the β-cell (the most important being; ER stress, inflammasome activation with subsequent β-cell-driven cytokine (e.g., IL-1β) production and NF-κB activation and nitroxidative/oxidative stress). Together, these may then initiate low-grade inflammation in the islet microenvironment (102, 103). In T1D, a cascade of inflammatory cytokines secreted from the immune cells leads to autoimmune destruction of β-cells (104). Cellular senescence may also be involved (105, 106). The overt loss of functional β-cell mass in T1D is thought, ultimately, to result from accelerated apoptosis (3). ER stress is a common upstream culprit in both T1D and T2D (102, 104). The unfolded protein response, which may lead to ER stress, on the one hand, and NMD on the other, mutually regulate each other in mouse and human tissues and cell lines (71). Thus, UPR transcripts may be NMD-specific targets (58).

The NMD plays a central role in the RUST mechanism to eliminate the PTC-containing transcript isoforms generated due to perturbed AS (107). The degree and magnitude of NMD activity differs among studied mice and human cells and tissues, as reviewed previously (89). In transgenic mice ubiquitously expressing the *Men1* gene, the ratio of PTC-containing versus wild-type transcripts was significantly different between adult mouse tissues. Among the tested tissues, testis, ovary, brain, and heart exhibited high NMD activity, measured by strong downregulation, and lung, intestine, and thymus exhibited weak downregulation of the mutant *Men1* transcripts compared to wild-type transcripts (108). Unfortunately, neither this study nor others reported NMD efficacy in the pancreas.

**Figure 4:**
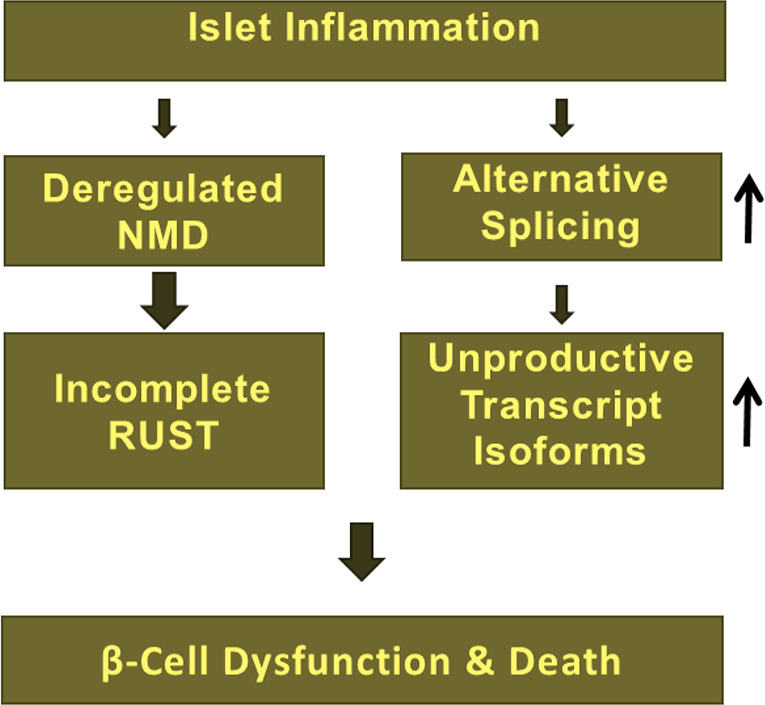
Deregulation of NMD due to islet inflammation largely influence RUST mechanism of transcript processing and subsequent accumulation of unproductive transcript isoforms, which implicates in β-cell dysfunction, vulnerability, and death.

Other studies have suggested that NMD efficiency varies among individuals with nonsense mutations e.g., in the cystic fibrosis gene *CFTR* in response to the drug PTC124, forcing read-through of mutated mRNAs (**Figure 2B**) (109). The RUST mechanism was first proposed by Lewis and colleagues in 2003 (64). These authors found that 35% (i.e., 1,989 out of 5,693) of alternatively spliced transcript isoforms in the human cell transcriptome were NMD targets since they contained PTCs (64). Several subsequent studies identified a role for incomplete RUST in regulating transcript processing in breast and myelodysplastic syndrome (MDS) cancers, and in neurological disorders such as Alzheimer’s disease and multiple sclerosis (16). However, the role of RUST is completely unknown in transcript processing in pancreatic β-cells. Knowing that pro-inflammatory cytokines regulate alternative splicing events and the NMD pathway in human and rodent primary β-cells (9, 28, 37, 40, 41), we propose a model (**Figure 4**) in which incomplete RUST leads to accumulation of unproductive transcripts whose translation into unfolded, truncated polypeptides overwhelms ER capacity and consequently drives unresolved ER stress. Consistent with this view, enforcing ribosomal read-through of such PTC-containing mRNAs with the drug PTC124 aggravates cytokine-induced apoptosis and is associated with an increase in ER stress in human islets and INS-1 cells (7).

## Perspective: A New Type of Unproductive Transcript?

The recent discovery of a special class of bifunctional RNAs, namely coding-noncoding RNAs (cncRNA), implicates another culprit in the pathogenesis of diseases such as Alzheimer’s disease. Amongst the cncRNAs, certain noncoding mRNA isoforms (ncimRNA) of (usually) protein-coding genes quantitatively predominate (110, 111). The precise function and underlying molecular mechanism(s) of action of cncRNAs has been investigated in a few cases (110). Insulin receptor substrate 1 (IRS1), a major substrate and cytoplasmic docking protein for the insulin receptor and insulin-like growth factor receptor, is involved in insulin signalling. The level of IRS1 is highly increased in proliferative cells such as human and mouse cancer cells (110), whereas profoundly decreased in differentiated cells. In addition, whole body *Irs1* knockout mice exhibited severe insulin resistance in skeletal muscle and liver, with compensatory β-cell hyperplasia (112). Surprisingly, a further study (113) found that the 5′UTR of *Irs1* mRNA acts as an antisense mRNA to the cell cycle regulator retinoblastoma (Rb).

Intronic or exonic Circular RNAs (circRNAs) are a type of single-stranded noncoding RNAs whose 5’ and 3’ termini are covalently linked by back-splicing of exons from a single pre-mRNA and they are, therefore, stable and resistant to exonuclease degradation (114). With a feature of cell-specificity and being conserved between species, circRNAs play important roles in the development of diseases by modulating post-transcriptional regulation of gene expression (114, 115). Recently, two intronic circRNA borne from murine insulin genes, ci-Ins2 and ci-INS have been reported to control insulin secretion. Thus, silencing of ci-Ins2 in pancreatic islets deceases in the expression of key components of the secretory machinery of β-cells, resulting in impaired pulsatile insulin secretion and calcium signalling (116). Interestingly, these circRNAs were shown to interact with the RNA-binding protein TAR DNA-binding protein 43 KDa (TDP43) (116), indicating a possible correlation with alternative splicing and pre-mRNA turnover, and eventually NMD activity.

## Discussion

Pancreatic β-cells must fine tune protein synthesis given large fluctuations between low and very high demands for insulin secretion. Post-transcriptional regulation plays an important role (33, 117). Transcript processing is, however, not limited only to alternative splicing events. Other homeostatic pathways including NMD directly, and UPR indirectly, are involved in this fine-tuning. Inflammatory cytokines and glucolipotoxicity are major drivers of ER stress in pancreatic β-cells leading to UPR and NMD activation and are likely needed to efficiently and accurately eliminate unproductive transcript isoforms (**Figure 2C**). If these remained intact, the production of truncated proteins may exert deleterious effects on β-cell function and viability.

With respect to the identified role of RBPs and alternative splicing factors in pancreatic β-cell function and viability, and given that over 90% of human genes transcribed into at least four transcript isoforms (17, 118–120), we suggest that transcript processing by the RUST mechanism may be mandatory to guarantee functional accuracy and integrity of pancreatic β-cells. In regard to the development, differentiation, function and resilience of pancreatic β-cells in health and in diabetes, we suggest that the following questions represent important areas for future research:

1. What is the role of the NMD in regulating normal transcripts?
2. Can we identify NMD-specific targets?
3. What is the contribution of the NMD to the RUST mechanism of eliminating unproductive transcript isoforms?
4. Do inflammatory and glucolipotoxic stresses exerts adverse effects on insulin biosynthesis and secretion, as well as cell viability, through changes in NMD activity?
5. If so, which branches of NMD are involved and are the key components indispensable in normal, stress and disease conditions?

## Author Contributions

The first draft was written by SG and edited by GR and SG. All authors contributed to the article and approved the submitted version.

## Funding

This study was supported within the independent postdoctoral grant (international mobility, grant number: 9034-00001B) for SMG by the Independent Research Fund Denmark (DFF-Medical Council). GR was supported by a Wellcome Trust Senior Investigator (WT098424AIA) and Investigator (WT212625/Z/18/Z) Awards, MRC Programme grants (MR/R022259/1, MR/J0003042/1, MR/L020149/1, MR/R022259/1) and Experimental Challenge Grant (DIVA, MR/L02036X/1), MRC (MR/N00275X/1), Diabetes UK (BDA/11/0004210, BDA/15/0005275, BDA 16/0005485), and Imperial Confidence in Concept (ICiC) grants. This project has received funding from the European Union’s Horizon 2020 research and innovation programme *via* the Innovative Medicines Initiative 2 Joint Undertaking under grant agreement No 115881 (RHAPSODY) to GR.

## Conflict of Interest

GR has received grant funding and consultancy fees from Sun Pharmaceuticals and Les Laboratories Servier for unrelated studies.

The remaining author declares that the research was conducted in the absence of any commercial or financial relationships that could be construed as a potential conflict of interest.

## Abbreviations

NMD, nonsense-mediated decay; AS, alternative splicing; PTCs, premature termination codons; T1D, type 1 diabetes; T2D, type 2 diabetes; RUST, Regulated Unproductive Splicing and Translation; SMD, Staufen1 (STAU1)-mediated mRNA decay; NGD, no-go decay; NSD, nonstop-decay; RBPs, RNA binding proteins; SREs, splicing regulatory elements; SRs, Serine Arginine rich proteins; hnRNP, heterogeneous nuclear ribonucleoprotein; ESE, exonic splicing enhancers; ISS, intronic splicing suppressors; IL-1β, interleukin-1β; IFNɣ, interferon-ɣ; NOD mice, Non-obese diabetic mice; HbA1c, haemoglobin A1c; TCF7L2, Transcription factor 7–like 2; Cask, calcium/calmodulin-dependent serine protein kinase; Madd, MAP kinase-activating death domain; uORF, upstream open reading frame; 3’-UTR, long 3’ untranslated region; eRF2, eukaryotic releasing factors 2; eRF3, eukaryotic releasing factors 3; EJC, exon-junction complex; PI3K, phosphoinositide 3-kinase; PNRC2, Proline-rich nuclear receptor co-activator 2; iNOS, inducible nitric oxide; NMA, N-methyl-L-arginine; MDS, myelodysplastic syndrome; ATAC-sequencing, assay for transposase-accessible chromatin with sequencing; cncRNA, coding-noncoding RNA; ncimRNA, noncoding mRNA isoforms; IRS1, Insulin receptor substrate 1; circRNA, circular RNA; TDP43, TAR DNA-binding protein 43 KDa; RB, retinoblastoma; Gsn, gelsolin; Cacna1c, calcium channel 1C; INSR, insulin receptor; PLC β1, Phospholipase Cβ1; SNAP-25, synaptosomal-associated protein 25; SNPs, Single nucleotide polymorphisms; UPF1-3, Up-Frameshift Suppressor 1 Homolog.
